# Acute haemodynamic decompensation in the era of substrate-based ablation: rare but still worrisome

**DOI:** 10.1093/europace/euae144

**Published:** 2024-06-12

**Authors:** Ioan Liuba, Christian De Chillou, Pasquale Santangeli

**Affiliations:** Section of Cardiac Pacing and Electrophysiology, Heart and Vascular Institute, Cleveland Clinic, Cleveland, 9500 Euclid Avenue, Cleveland, 44195 OH, USA; Department of Cardiology, University Hospital Nancy, Nancy, France; Section of Cardiac Pacing and Electrophysiology, Heart and Vascular Institute, Cleveland Clinic, Cleveland, 9500 Euclid Avenue, Cleveland, 44195 OH, USA

**This editorial refers to ‘Periprocedural acute hemodynamic decompensation during substrate-based ablation of scar-related ventricular tachycardia: a rare and unpredictable event’ by P. Stojadinović *et al*. https://doi.org/10.1093/europace/euae145.**

Ablation of ventricular tachycardia (VT) in patients with structural heart disease (SHD) has proved effective in reducing arrhythmia burden and the number of appropriate ICD shocks.^1,2^ The rate of periprocedural complications varies with the severity of the underlying cardiomyopathy, and the burden of associated comorbidities.^3,4^

Periprocedural acute haemodynamic decompensation (AHD) is a particularly severe complication, associated with excess mortality after different types of endovascular interventions and surgical procedures.^5–7^ The initial evidence supporting the incidence and prognostic implications of AHD associated with VT ablation in SHD was provided by a report from the University of Pennsylvania in 2015.^8^ In an observational cohort of 193 consecutive patients with SHD who underwent VT ablation, it was found that periprocedural AHD, defined as persistent hypotension refractory to vasopressors and resulting in emergent placement of mechanical haemodynamic support and/or procedure discontinuation, occurred in 11% of the patients. It was further observed that AHD was associated with substantial post-procedural mortality rate. Thus, at a mean follow-up of 21 months, the all-cause mortality was 50% patients who developed AHD compared to 11% of those without AHD. These sobering data substantiated the existing awareness regarding the deleterious effects of AHD and the potential benefit of pre-emptive measures, such as mechanical circulatory support in these cases.^9,10^ Furthermore, they highlighted the value of upfront risk stratification tools with the aim of early identification of high-risk patients and appropriate pre-procedural planning to improve prognosis.

The same report identified eight variables predicting periprocedural AHD: age > 60 years, diabetes mellitus, ischaemic cardiomyopathy, left ventricular ejection fraction < 25%, chronic obstructive pulmonary disease, presentation with VT storm, and NYHA functional class ≥ III. General anaesthesia was found to be an additional risk variable. Based on these findings, the PAINESD risk score was built by incorporating all these variables. A prediction model based on tertiles of the PAINESD score was derived to identify patients at lowest and highest risk of AHD. The risk of AHD increased across tertiles, with patients in the highest tertiles (≥17 when including general anaesthesia as a variable) reaching an incident risk of 24%.

An acknowledged limitation of the PAINESD risk score is that it has been derived from an observational study, and hence subject to selection bias. It was also derived from a non-contemporary cohort of patients from more than 10 years ago, and procedural techniques have since then evolved significantly with more systematic implementation of substrate-based ablation approaches with minimal VT induction. However, the performance of PAINESD score in predicting AHD has been subsequently confirmed in multiple independent cohorts.^11,12^

In this issue of the journal, Stojadinović *et al*.^13^ report the results of a retrospective study assessing the incidence of AHD in a cohort of patients who underwent catheter ablation of scar-related VT over a period of 14 years at the author’s institution. A total number of 1124 patients were included (67% ischaemic cardiomyopathy, mean left ventricular ejection fraction = 34%, NYHA class: 2 ± 1, 25% patients presented with electric storm, mean PAINESD score: 11.4 ± 6.6). The vast majority of procedures (85%) was performed under conscious sedation except for cases with planed epicardial access and patients with VT storm and secondary haemodynamic instability. A primarily substrate-based ablation approach was employed. The key findings were: (1) a total of 13 out of 1124 patients, representing 1.2% of the entire cohort, developed AHD, defined as development of acute pulmonary oedema or refractory hypotension requiring intervention, including inotropic agents, artificial ventilation, or implantation of a mechanical haemodynamic support device; (2) a PAINESD stratification by tertiles did not predict the occurrence AHD; and (3) a total number of 539 patients (48%) died over a mean follow-up of 4.2 years. The PAINESD score was found to be a strong predictor of all-cause mortality. On multivariate logistic regression, the following variables were independent predictors of death: age > 60 years, diabetes, ischaemic cardiomyopathy, left ventricular ejection fraction < 25%, presentation with VT storm, and NYHA functional class ≥ III.

The author’s group, recognized for their expertise in the field of VT ablation, is to be commended for reporting their experience on a large series of patients. Overall, the study is consistent with the available body of evidence regarding the value of PAINESD score in predicting post-procedural adverse events (including early mortality), although it did not predict periprocedural AHD, which is possibly due to the small number of events and lack of statistical power to detect difference between groups. In addition, the incidence of AHD and the predictive performance of the PAINESD reported by Stojadinović *et al*.^13^ should be interpreted in the context of the clinical characteristics and procedural approaches adopted in their study population. It is noteworthy that the vast majority of patients presented in sinus rhythm (and not in incessant/high burden of VT) and that ablation was performed mainly via substrate-based techniques. General anaesthesia was reported in 15% of cases. For comparison, in the prior study from the University of Pennsylvania group,^8^ the procedural approach relied on both mapping of haemodynamically stable VTs and substrate-based approaches for unstable VTs, targeting both clinical and non-clinical VTs, mainly guided by results of repeat programmed stimulation to assess the procedural success defined by lack of inducibility. None of the patients underwent a pure substrate-only ablation approach, and a median of 2 VTs/patient were induced and attempted to map during the procedure. In addition, general anaesthesia was used in one-third of the patients (32%). Taken together, these findings suggest that the differences in AHD rate between that reported by Stojadinović *et al*. and that reported by the prior study by Santangeli and colleagues are likely related to differences in patient population and, most importantly, procedural approaches.

As mentioned, the low rate of AHD occurrence reported by Stojadinović *et al*. might explain the lack of differences across the PAINESD tertiles, as also indicated by the marginal statistical significance between the average PAINESD score values in patients who developed AHD compared to those without AHD (14.9 ± 9 vs. 11.4 ± 6.6, *P* = 0.05).

Importantly, the PAINESD score was a strong predictor of post-procedural mortality, for which much more events were available for analysis compared to AHD, hence improving statistical power to detect differences. Of note, all variables included in the PAINESD score except for chronic obstructive pulmonary disease were found to be independent predictors of mortality following ablation, which is also consistent with previous studies.^12,14,15^

Lastly, it is it is important to stress that, despite the benefit of substrate ablation to prevent AHD events, this approach is not feasible in important patient subsets, such as those presenting with high intraprocedural VT burden and patients with non-ischaemic cardiomyopathy and predominant intramural scar, in whom it may be difficult to identify critical ablation sites with mapping in sinus/paced rhythm. In these cases, VT induction and activation mapping is often necessary in order to disclose critical segments of the arrhythmogenic substrate to be then targeted with ablation.^16,17^ As such, risk stratification tools and pre-procedural identification of patients with non-ischaemic cardiomyopathy at high risk for developing AHD might be of great importance, as it allows for careful planning of the ablation approach to prevent haemodynamic instability associated with multiple VT inductions.^18^

In conclusion, the investigation by Stojadinović adds important information to previous research in the field of VT ablation in patients with SHD, and indicates that periprocedural AHD may be mitigated during scar-related VT ablation by predominant substrate ablation techniques with minimal use of general anaesthesia. However, we believe that the PAINESD score remains an important tool to identify patients at higher risk post-procedural mortality, as the present study by Stojadinović confirms, as well as potential for periprocedural AHD, particularly in patients with limited or elusive substrate that require VT induction for mapping and/or anticipated high burden of spontaneous intraprocedural VT.^8,12,13^
